# Epicardial Abnormalities and Mesenchymal/Hematopoietic Cell Expansion in Plakophilin 2-Null Mouse Embryonic Hearts

**DOI:** 10.3390/cells14221751

**Published:** 2025-11-08

**Authors:** Mistura Dolapo Bolaji, Pia E. Hartmann, Eva Miriam Buhl, Robin M. W. Colpaert, Francesca Gasparella, Leon J. de Windt, Martina Calore, Rudolf E. Leube, Hoda Moazzen

**Affiliations:** 1 Institute of Molecular and Cellular Anatomy, RWTH Aachen University, Wendlingweg 2, 52074 Aachen, Germany; mbolaji@ukaachen.de (M.D.B.); rleube@ukaachen.de (R.E.L.); 2 School of Cardiovascular Disease (CARIM), Faculty of Health, Medicine & Life Sciences (FHML), Maastricht University, Universiteitssingel 50, 6229ER Maastricht, The Netherlands; pia@hartmann-pool.de (P.E.H.); r.colpaert@uke.de (R.M.W.C.); l.dewindt@maastrichtuniversity.nl (L.J.d.W.); or martina.calore@unipd.it (M.C.); 3 Electron Microscopy Facility, Institute of Pathology, RWTH Aachen University Hospital, Pauwelsstrasse 30, 52074 Aachen, Germany; ebuhl@ukaachen.de; 4 Department of Biology, Padova University, via Ugo Bassi 58B, 35131 Padova, Italy; francesca.gasparella@phd.unipd.it

**Keywords:** desmosome, plakophilin 2, hematopoiesis, hemopericardium, embryonic heart, heart development, intercellular junctions

## Abstract

Desmosomal junctions provide structural stability supporting concerted cardiomyocyte contractility. Previously, we demonstrated that a deficiency in the desmosomal transmembrane cadherin desmoglein 2 (Dsg2) reduces desmosome formation and disrupts cardiac morphogenesis, leading to excessive endothelial-to-hematopoietic cell transformation and embryonic lethality. It remained unclear whether this phenotype was specifically driven by Dsg2-deficiency or was a broader consequence of impaired desmosome adhesion. To address this question, we generated *Pkp2^mt^*^/*mt*^ mouse embryos lacking the desmosomal plaque protein Pkp2, which resulted in loss of desmosome formation. Despite the absence of cardiac wall rupture, *Pkp2^mt^*^/*mt*^ and some *Pkp2^wt^*^/*mt*^ presented accumulations of Ter-119^+^ blood cells and RUNX1^+^/CD44^+^ hematopoietic stem cells in the pericardial space. Remarkably, in *Pkp2^mt^*^/*mt*^ hearts, the epicardium was detached from the myocardium, contained rounded cells expressing the hematopoietic stem cell marker RUNX1, and showed altered intermediate filament expression. These findings suggest a potential trans-differentiation of the epicardial cells into hematopoietic cells. In conclusion, deficiencies in both Dsg2 and Pkp2 promote hematopoiesis in the developing murine heart but target different cell types, i.e., endothelial cells, which lack desmosomes, or desmosome-containing epicardial cells. Our results provide evidence for the involvement of Pkp2 in epicardial morphogenesis and remodeling.

## 1. Introduction

The sophisticated heart structure emerges through the coordinated contributions of cardiomyocytes, endocardial cells, and epicardial cells. Anatomically, epicardial cells cover the outer surface of the myocardium, while endocardial cells line its inner surface. Endocardial cells have limited direct physical contact with cardiomyocytes, as extracellular matrix forms a crucial interface between these layers. Epicardial cells initially form adhesions with cardiomyocytes, and the extracellular matrix will be deposited later between the two cell layers to seal them together. Epicardial and endocardial cells both play distinct yet complementary roles in heart development and maturation. Epicardial cells contribute to myocardial growth by providing interstitial fibroblasts and smooth muscle cells, whereas endocardial cells regulate myocardial maturation, septation, and valve formation. An important feature of both cell types is their ability to undergo epithelial-to-mesenchymal transition (EMT), allowing them to remodel the embryonic heart and support its functional development [1,2].

In the developing heart, the cell membranes of immature cardiomyocytes are first connected by N-cadherin junctions, followed by the formation of desmosomal junctions across all cell borders [3,4]. As cardiomyocytes mature, desmosomal junctions become increasingly abundant and organized within polarized intercalated disks, providing a critical structural platform for the continuous contraction cycle of cardiomyocytes [5,6]. Desmosomes are also pivotal in early stages of heart development, as evidenced by the mortality of murine embryos lacking desmosomal proteins such as plakoglobin, desmoplakin, and plakophilin 2 (Pkp2). These proteins, in addition to desmosomal cadherins desmoglein 2 (Dsg2) and desmocollin 2, build the core unit of desmosomes. Notably, mutations in each of these components disrupt heart development with variable penetrance [7,8,9,10]. The most striking and common cardiac abnormality is hemopericardium, i.e., accumulation of blood cells within the pericardial cavity, which is observed in all desmosomal mutant murine models [6]. The cause of the observed cardiac abnormalities remained enigmatic for a long time until we could show that a deficiency in the desmosomal cadherin desmoglein 2 (Dsg2) leads to endocardial cell transformation into proliferating and differentiating hematopoietic stem cell clusters within the myocardial wall, which were either resorbed or caused myocardial rupture and subsequent death of the embryo at mid-gestation [11]. It remained unclear, however, whether this phenotype was specific for Dsg2-depletion in endocardial cells independent of desmosome function or a consequence of desmosome disruption in cardiomyocytes. Given the critical role of Pkp2 in desmosome formation [9,12], we aimed to investigate this question using a murine *Pkp2*-mutant mouse model. Previous work by Grossmann et al. (2004) attributed the observed hemopericardium in *Pkp2*-deficient embryos to blood cell leakage caused by compromised intercellular junctions between cardiomyocytes [9]. However, direct evidence for cardiac wall rupture was not presented. Even more, the underlying cellular mechanisms remained unresolved. Notably, the study did not assess the development of endocardial and epicardial cells and their possible contribution to the observed dysfunction.

In humans, mutations in desmosomal genes are associated with arrhythmogenic cardiomyopathy (AC), a disease characterized by cardiomyocyte death and replacement with fibrotic and fatty tissue [13]. The progression of AC is thought to result from a combination of mechanical instability and disrupted molecular signaling within cardiomyocytes [14,15]. Emerging evidence suggests that epicardial-derived cells may play a significant role in disease progression [16]. Epicardial cells hold a special value in cardiac pathobiology, as they can modulate fibrotic, vascular, and inflammatory remodeling of an injured heart [6,17]. While substantial progress has been made in understanding the complex pathophysiology of AC in the adult heart, the interplay between different cell types during cardiogenesis remains less explored.

In the present study, we conducted a comprehensive histological and molecular analysis to investigate the role of Pkp2 in cardiac morphogenesis using *Pkp2*-mutant (*Pkp2^mt^*^/*mt*^) mice, which only express a severely truncated 79 amino acid-long, non-membrane-localized form of Pkp2 and lack desmosomes in the developing heart. Our immunohistochemical analysis revealed that epicardial cells fail to develop normally in homozygous mutants. They round up and lose their attachment to the underlying myocardium and are replaced by hematopoietic stem cells, as indicated by RUNX1 expression. The hematopoietic stem cells subsequently differentiate into erythrocytes and accumulate either in the pericardial space or subepicardium. Consequently, the number of epicardial cells covering the myocardium is significantly reduced, and myocardial mass is compromised, leading to death at embryonic day (E) 11.5. Our data, together with previous observations [11], indicate that desmosomal proteins are crucial for maintaining cardiac tissue homeostasis and suppressing excessive cell fate transitions.

## 2. Materials and Methods

### 2.1. Generation and Genotyping of the Pkp2 Mutant Mouse Model

The Pkp2^mt^ mouse model was generated with the help of CRISPR-Cas9 technology using FVB/NRj zygotes. The premature stop codon at arginine 79 (TCACCG to TCATTA) was inserted through homology-directed repair, resulting in a non-functional amino-terminal Pkp2 fragment. Genotyping was performed via PCR using primers GCATACACTACTGGCACTTGG (forward) and CTGCCGTCCAACAAAGTCAT (reverse), producing a 273 bp amplicon. Subsequent HphI restriction enzyme digestion (New England Biolabs (Ipswich, MA, USA), R0158S) distinguished wild-type and mutant alleles: the wild-type amplicons were cleaved into 193 bp and 80 bp fragments, whereas the mutant amplicons remained uncut. Pkp2 protein levels were assessed in adult hearts by immunoblot analyses. Hearts were snap frozen and lysed in a modified RIPA buffer (50 mM Tris-HCl pH 7.5, 150 mM NaCl, 10 mM MgCl_2_, 1 mM EDTA pH 8.0, 10% glycerol, 1% Triton X-100, 0.5 mM dithiothreitol, 2% sodium dodecyl sulfate) containing protease and phosphatase inhibitor cocktails (Roche Diagnostics, Rotkreuz, Switzerland). Protein concentration was determined after centrifugation at 18,000× *g* for 10 min at 4 °C in the soluble fraction by Pierce BCA Protein Assay (ThermoFisher Scientific (Waltham, MA, USA), 23227). A 4 × loading buffer (ThermoFisher Scientific, NP0007) and 10× sample-reducing agent (ThermoFisher Scientific, NP0009) were added to 30 µg of protein lysate, which was denatured at 95 °C for 5 min and resolved in 4–12% Bis-Tris gels (ThermoFisher Scientific, NP0322BOX). Proteins were transferred to a nitrocellulose membrane and subsequently blocked with 5% nonfat milk. The membrane was incubated with primary antibody against Pkp2 (Progen (Heidelberg, Germany), 651101; 1:100 in 2.5% milk PBST) overnight at 4 °C and then with secondary antibody (ThermoFisher Scientific, 31430; 1:10,000 in 1% milk PBST) for 1 h. Vinculin (ThermoFisher Scientific, mA5-11690; 1:1000 in 2.5% milk TBST) served as loading control. Signal detection was performed by ECL (ThermoFisher Scientific, 34577) using a UVITEC Cambridge Mini HD imaging system (UVItech, version 17.01). Quantitative analysis was performed by the ImageJ software (version 1.54p).

### 2.2. Embryo Collection, Histology, Immunohistochemical Assays, and Microscopy

Heterozygous *Pkp2^mt^*^/+^ mice were bred to generate *Pkp2^mt^*^/*mt*^ embryos. Embryos were collected at E9.5, E10.5, E11.5, and E12.5 at midday following cervical dislocation of pregnant dames. For visualization of the Krt8-YFP, transgenic mice were used to visualize keratin filaments in the epicardium of Krt8-YFP mice. These animals express fluorescently tagged simple epithelial keratin 8 from its endogenous gene locus [18]. The epicardium was isolated from these mice to visualize the abundant keratin filament networks in this tissue. All animal procedures were performed in accordance with the recommendations of the Guide for the Care and Use of Laboratory Animals. The use of *Pkp2* mice was approved by Medanex EC Code: EC MxCl 2019-136, on 14.02.2020 and the use of *Krt8-YFP* mice was approved by the institute for Laboratory Animal Science, RWTH Aachen, Germany, reference number: 50182 A4 notifications for killing animals for scientific purposes, on 20.07.2022. The study is reported in accordance with ARRIVE guidelines.

Genotyping was carried out on tail biopsies, while the upper body was fixed overnight in 4% formaldehyde. Tissues were dehydrated through a graded isopropanol series and embedded in paraffin. Whole embryos were sectioned at 5 μm thickness for histological analysis using hematoxylin/eosin staining or for immunoanalysis. Immunofluorescence labeling and immunohistochemistry were performed as previously reported [11]. Briefly, following deparaffinization and rehydration, antigen retrieval was accomplished in 10 mM citrate buffer (pH 6) at 121 °C for 3 min. Primary antibody was diluted and incubated on tissue sections overnight in 1.5% (*w*/*v*) bovine serum albumin (BSA) in PBS at 4 °C, followed by washing in Tris/HCl buffer (pH 7.5). Immunofluorescence staining was performed by incubation of samples with secondary antibodies for one hour at room temperature. Nuclear counterstaining was performed with 2 μg/mL DAPI in PBS for 30 min before mounting. For immunohistochemistry, color detection of rat and rabbit primary antibodies was achieved using the Histofine Simple Stain Mouse MAX PO (Rat)-Kit (Nichirei Biosciences Inc., Tokyo, Japan) and the ZytoChem Plus (HRP) Polymer Kit (ZytoVision GmbH, Bremerhaven, Germany), respectively, followed by subsequent 3,3′-diaminobenzidin (DAB) staining and hematoxylin counterstaining. A complete list of antibodies and their specific dilutions is provided in Supplementary Table S1 (see Table S1).

Light and fluorescence microscopic images were recorded with Axiophot and ApoTome.2 imaging systems (both from Zeiss, Oberkochen, Germany) and processed by Zen 2 and Zen 3.3 software (blue edition; https://www.zeiss.com/microscopy/en/products/software/zeiss-zen.html#related-products accessed on 5 May 2025), respectively.

### 2.3. Electron Microscopy

Samples were fixed in 3% glutaraldehyde and embedded in 2.5% low-melting agarose (Sigma, St. Louis, MO, USA). After post-fixing with 1% OsO_4_ (Roth, Bavaria, Germany), the samples were dehydrated by ascending ethanol series, then in propylene oxide (Science Services, Munich, Germany), and they were then embedded in Epon. Ultrathin sections were stained with 0.5% uranyl acetate and 1% lead citrate (both Science Services) and examined using a transmission electron microscope (Zeiss Leo906).

### 2.4. Cell Culture and Immunofluorescent Analysis

Healthy control hiPSCs (line 106) were generously provided by Wolfgang Wagner (Uniklinik RWTH Aachen, Aachen, Germany). hiPSCs were grown on Geltrex (ThermoFischer, A1413301) and maintained in E8 medium (ThermoFisher, A1517001), which was refreshed daily. Differentiation to epicardial cells was carried out using previously published protocols [19]. Briefly, 125,000 hiPSCs were seeded on a 12-well plate to reach confluency over three days in E8 medium (day −3 to day 0). Mesoderm differentiation was initiated by adding 10 μM of CHIR99021 (Sigma-Aldrich (St. Louis, MO, USA), 361559) in albumin-containing RPMI 1640 (ThermoFisher, 72400-021) plus B27 supplement minus insulin (Life Technologies, Carlsbad, CA, USA). This was considered day 0. The medium was changed after 24 h. On day three, 5 μM IWP2 (Sigma-Aldrich, 681671) was added, and the medium was changed again on day 5. On day 6, the cells were reseeded onto 0.1% gelatin-coated dishes in Advanced DMEM/F12 (ThermoFischer, 12634010) with 2.5 mM GlutaMAX, 1% fetal bovine serum, and 100 μg/mL ascorbic acid. This medium was called LaSR medium. On day 7, the cells were treated with 3 μM CHIR99021 in LaSR medium. By day 14, the cells demonstrated morphological and molecular markers of epicardial cells. The cells were maintained in LaSR medium and received 0.5 μM A83-01 (Sigma-Aldrich, SML0788), an inhibitor of TGF-β signaling. For immunoanalysis of protein expression, cells were expanded on 0.1% gelatin-coated coverslips to reach confluency, then cells were fixed in 4% paraformaldehyde (PFA) for 20 min at room temperature. Coverslips were washed and incubated with diluted primary antibody for 1 h at room temperature. Following two washes, the diluted secondary fluorescent antibody containing 2 μg/mL DAPI for nuclear counterstaining was incubated on the samples for 1 h. Coverslips were washed with PBS and mounted on a glass slide for further microscopical visualization. The list of antibodies and their dilution is provided in Supplementary Table S1 (Table S1) [20].

## 3. Results

### 3.1. Pkp2 Deficiency Induces Embryonic Mortality at E11.5

We generated a nonsense c.235C>T (p.R79*) mutation in the *Pkp2* gene, which was based on the reported mutation in AC patients ([21]; Figure S1A). The embryos were genotyped, confirming the successful deletion of the targeted sequence (Figure S1B). Adult *Pkp2* heterozygous hearts demonstrated a slight reduction in Pkp2 protein levels (Figure S1C). Mating of heterozygous *Pkp2^mt^*^/*wt*^ animals produced no live *Pkp2^mt^*^/*mt*^ offspring, indicating that the homozygous *Pkp2* mutation is embryonically lethal, as expected from the comparable genetic mutation in Grossmann et al. [9]. To determine the time point of death, embryos were harvested at E9.5, E10.5, E11.5, and E12.5 (*N* = 109 embryos collected from 13 females, Table 1). All *Pkp2^mt^*^/*mt*^ embryos were alive at E9.5 and E10.5. By E11.5, however 90%, of *Pkp2^mt^*^/*mt*^ embryos were dead as determined by their pale color or resorbed morphology. No live *Pkp2^mt^*^/*mt*^ embryos could be retrieved at E12.5. These findings suggest a critical window of mortality occurring shortly after E10.5. This observation was in agreement with the report of Grossmann et al. [9], but the mortality was more penetrant and occurred earlier than in *Dsg2*-mutant embryos [11].

### 3.2. Desmosomes Are Absent in Pkp2^mt/mt^ Hearts

**Figure 1:**
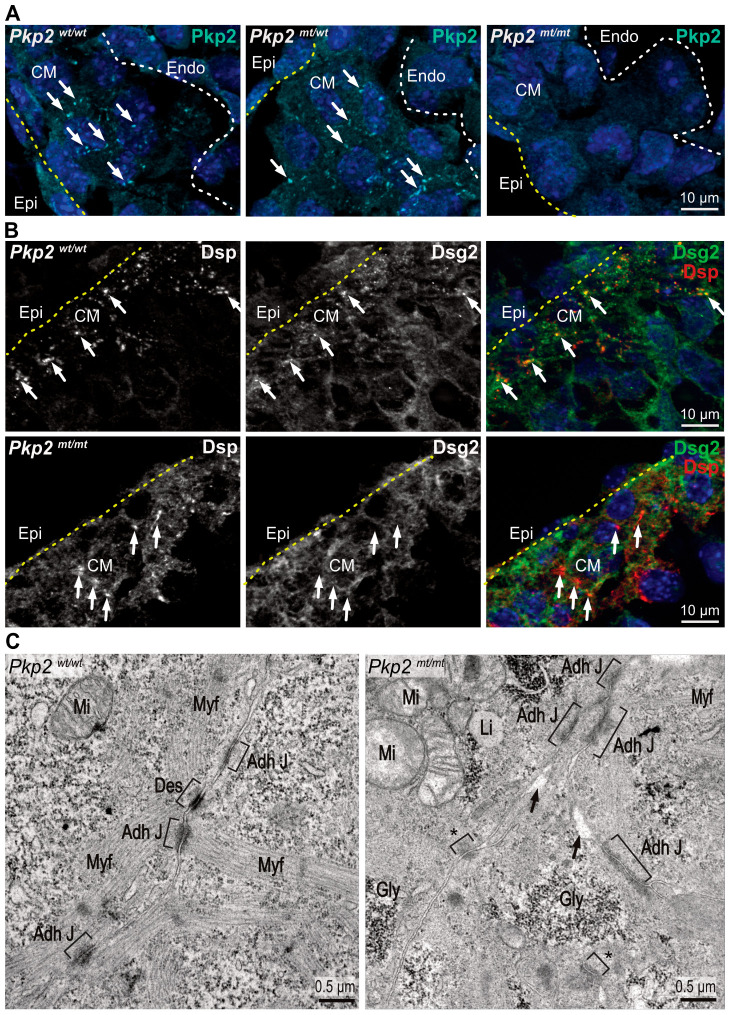
*Pkp2^mt^*^/^*^mt^* myocardium lacks bona fide desmosomes. Visualization of desmosome formation by immunofluorescence microscopy of desmosomal components (**A**,**B**) and by transmission electron microscopy (**C**) in ventricular myocardium. (**A**) The fluorescence images show that Pkp2 localizes to distinct puncta at plasma membrane sites of adjacent cardiomyocytes in E10.5 wild-type myocardium. Reduced expression is observed in heterozygous *Pkp2^mt^*^/^*^wt^* samples and a complete loss of Pkp2 expression in homozygous *Pkp2^mt^*^/^*^mt^* hearts. Nuclei are stained with DAPI. Arrows indicate membrane-localized signals. Yellow dashed lines marks epicardium and white dashed line marks endocardial cells. *N* = 3–4 hearts per group. At least six heart sections and nine images were analyzed per individual heart. (**B**) The fluorescence micrographs show that anti-desmoplakin (Dsp) and anti-desmoglein 2 (Dsg2) immunofluorescence colocalize at defined plasma membrane sites in the wildtype. In contrast, Dsp staining looks diffuse while retaining some of the puncta, and Dsg2 does not enrich with Dsp in the remaining Dsp puncta and appears diffuse in *Pkp2^mt^*^/^*^mt^* mutant myocardium. *N* = 3 heart samples per group, at least six heart sections and 10 images were analyzed per heart. (**C**) The electron micrographs depict the ultrastructure of ventricular cardiomyocytes. In the wildtype, junctions are formed as expected, whereas typical desmosomal junctions are missing in the *Pkp2^mt^*^/^*^mt^* background. *N* = 2 embryos per group. Asterisks mark small focal adhesion sites that may correspond to immature junctions. Note the less organized sarcomeres in mutants. Square brackets mark the membrane junctions, and arrows point to intercellular space. CM: Cardiomyocytes; Adh J: Adherens Junction; Des: Desmosome; Myf: Myofilaments; Mi: Mitochondria, Li: Lipid droplets; Gly: Glycogen.

We examined the expression and localization of Pkp2 during cardiogenesis. Pkp2 immunofluorescence revealed a punctate, membrane-localized expression pattern at E9.5 and E10.5 in the wildtype (Figure S2A and Figure 1A). Reduced expression levels of Pkp2 in *Pkp2^mt^*^/^*^wt^* and its complete absence in *Pkp2^mt^*^/^*^mt^* embryos validated the null mutation model. We then examined the expression of Dsp and Dsg2 to evaluate the formation of desmosomal junctions. Anti-Dsp and anti-Dsg2 positive desmosomal junctions appeared at E9.5 in distinct puncta in the wildtype (Figure S2B) and became more abundant at E10.5 (Figure 1B). A reduced Dsp/Dsg2 co-localization signal was observed in *Pkp2^mt^*^/^*^wt^* mutant hearts (Figure S2B). A complete loss of membrane-localized Dsp and Dsg2 immunosignals was noted in *Pkp2^mt^*^/^*^mt^* embryos at E9.5 (Figure S2B) and E10.5 (Figure 1B). As expected, no colocalization signal could be detected for Pkp2 and Dsg2 in *Pkp2^mt^*^/^*^mt^* embryos (Figure S2C). The formation of N-cadherin junctions seemed not to be disturbed across all genotypes (Figure S3).

We then evaluated the formation of desmosomal junctions in electron micrographs. While desmosomes were readily identified in ventricular cardiomyocytes of wildtypes, we could not detect typical desmosomal junctions in *Pkp2^mt^*^/^*^mt^*. But *Pkp2^mt^*^/^*^mt^* cardiomyocytes formed focal junctions with cytoplasmic tufts of fluffy material. The mutant cardiomyocytes also presented disorganized myofilament fibers (Figure 1C). The absence of Pkp2 expression, along with the loss of Dsp-Dsg2 colocalization and the absence of desmosomal ultrastructures, shows that the *Pkp2^mt^*^/^*^mt^* animals serve as a model for desmosome-deficiency in the developing heart, as has been suggested previously [9,12]. 

### 3.3. Erythroid and Hematopoietic Stem Cells Accumulate in the Pericardial Cavity of Pkp2^mt/mt^ Embryos

**Figure 2:**
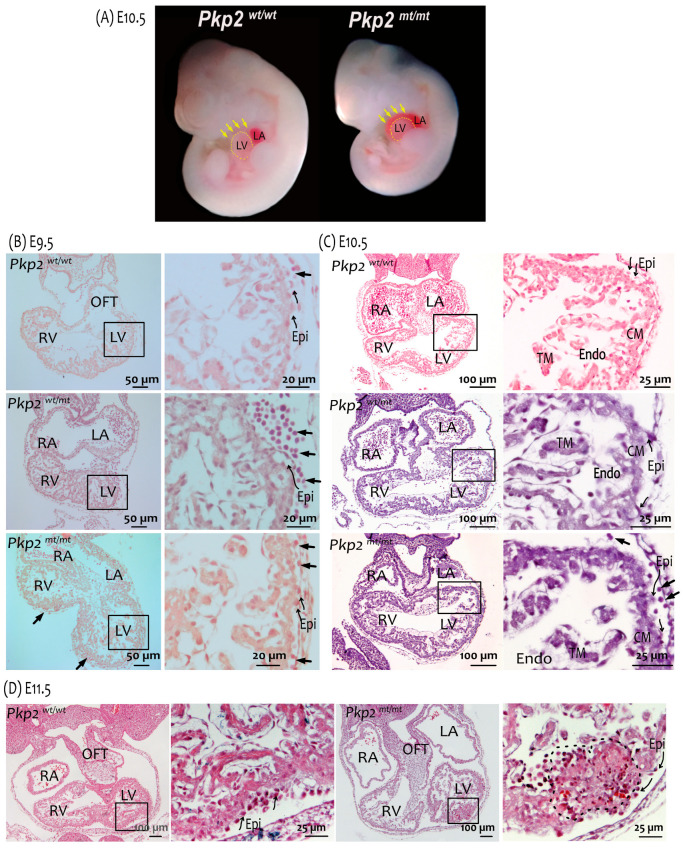
*Pkp2^mt^*^/*mt*^ hearts are characterized by hemopericardium, abnormal epicardium, and myocardial hypoplasia. (**A**) Gross anatomy of embryos shows a reduction in body size and the presence of hemopericardium (arrows) in *Pkp2^mt^*^/*mt*^ at E10.5. The border of the left ventricle (LV) is marked by a dashed line. Samples are imaged at the same magnification. (**B**–**D**) Images show comparisons of hematoxylin-eosin-stained tissue sections of wildtype, *Pkp2^mt^*^/*mt*^ and *Pkp2^mt^*^/*mt*^ embryonic hearts at E9.5, E10.5, and E11.5. (**B**) Short arrows point to circulating cells in the pericardial cavity of E9.5 embryos. They are rare in wildtypes and excessive in heterozygous and homozygous mutants. The curved arrows point to the epicardium (Epi). (**C**) Blood cells are consistently present in the pericardial cavity of E10.5 *Pkp2^mt^*^/*mt*^ embryos (arrows). Signs of abnormal epicardial development are visible in the mutant embryos and marked by curved arrows. (**D**) The myocardial wall is hypoplastic in *Pkp2^mt^*^/*mt*^ E11.5 hearts, and the tissue structure is disrupted (black dashed line). The organization and arrangement of epicardial cells are massively perturbed in the mutant heart (note curved arrows). Boxed areas in (**B**–**D**) are enlarged. Number of samples analyzed is reported in Table 1. CM, compact myocardium; TM, trabecular myocardium; Endo, endocardium; OFT, outflow tract; LV, left ventricle; RV, right ventricle; RA, right atrium; LA, left atrium.

**Figure 3:**
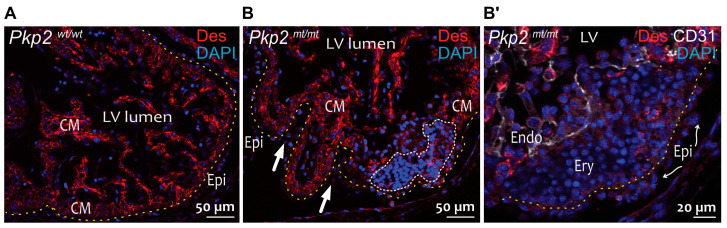
Non-cardiomyocyte cell clusters occur in the myocardium of E11.5 *Pkp2^mt^*^/^*^mt^* embryos. The fluorescence micrographs show the immunoreaction of anti-desmin antibodies detecting cardiomyocytes together with anti-CD31 antibodies reacting with endothelial cells (Endo) and nuclear DAPI staining. Desmin marks cardiomyocytes in wild-type (**A**) and *Pkp2^mt^*^/^*^mt^* hearts (**B**). CD31 marks endocardial cells in (**B′**). (**B**,**B′**) are parts of the same heart but in different sections. A yellow dashed line marks the basal aspect of epicardial cells. The white dashed line marks non-cardiomyocyte cluster in the myocardium. Blood cells (Ery, erythrocytes) are seen between the epicardial and endocardial cells within the myocardium of *Pkp2^mt^*^/^*^mt^* hearts. Arrows in B point to the deformed myocardial wall. Curved arrows point to epicardial cells (Epi) with loss of squamous morphology. LV: Left ventricle; CM: Cardiomyocytes. *N* = 2 hearts per group, at least 6 heart sections were analyzed per sample.

Histological analysis of *Pkp2^mt^*^/^*^mt^* embryonic hearts revealed normal cardiogenesis in terms of heart looping and chamber formation up to E10.5. However, hemopericardium, i.e., blood cells within the pericardial cavity, was observed in most *Pkp2^mt^*^/^*^mt^* embryos and, to some extent, in *Pkp2^wt^*^/^*^mt^* embryos at E9.5 and E10.5 (Figure 2A–C). At E10.5, hemopericardium was detectable in approximately 90% of *Pkp2^mt^*^/^*^mt^* embryos and 16% of *Pkp2^wt^*^/^*^mt^* embryos. Myocardial rupture was not seen in any of the sections, confirming the previous report of Grossmann et al., 2004 [9]. At E11.5, most of the embryos were dead, and the resorbed tissue could not be analyzed histologically anymore. The very few that survived showed hypoplastic myocardium with disruption of cardiomyocyte integrity and altered epicardial organization (Figure 2D). Desmin-positive cardiomyocytes were disorganized, and the structure of the myocardium was compromised (Figure 3). Although the heart is not vascularized at this stage, erythrocytes expanded massively in the subepicardium up to the endocardial cell layer in *Pkp2^mt^*^/^*^mt^* myocardium, disrupting myocardial integrity (Figure 3). This observation was unexpected and was not reported previously [9].

**Figure 4:**
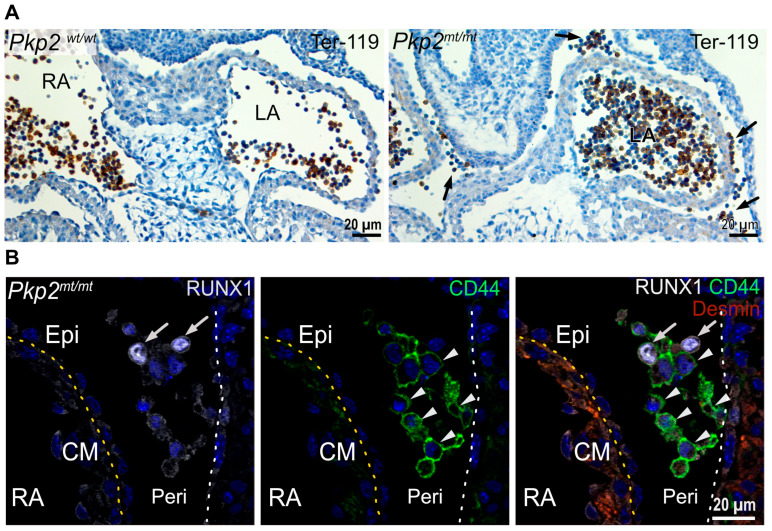
Floating erythropoietic and hematopoietic stem cells accumulate in the pericardial cavity of *Pkp2^mt^*^/^*^mt^* hearts at E10.5. (**A**) The microscopy images show cardiac tissue sections after immunohistochemical detection of Ter-119 and hematoxylin counterstaining. Note the abundance of Ter-119^+^ erythroid cells in the circulating blood of the left and right atrium (LA and RA, respectively) in the wild-type and mutant heart, whereas Ter-119^+^ cells are also present in the pericardial space of the *Pkp2^mt^*^/^*^mt^* hearts (arrows). (**B**) The fluorescence micrographs show immunodetection of the hematopoietic stem cell markers RUNX1 (arrow) and CD44 (arrowheads) in the pericardiac cavity of *Pkp2^mt^*^/^*^mt^* hearts. Desmin marks the myocardium. Epicardium and pericardial wall are marked by dashed yellow and white lines, respectively. Nuclei are stained with DAPI. *N* = 3–4 hearts per group, 3–6 heart sections were analyzed per sample. Epi, epicardium; CM, cardiomyocytes; Peri, Pericardium; RA, right atrium; LA, left atrium.

The cells in the pericardial cavity were heterogeneous with a large Ter-119^+^ erythroid cell population (Figure 4A; 194 Ter-119^+^ cells out of 565 cells in the pericardium, *N* = 2 embryos, 19 images were analyzed, which were obtained from at least six sections per heart). A subset of cells was positive for the hematopoietic stem cell markers RUNX1 and CD44 (Figure 4B, 9 RUNX1^+^ cells out of 347 cells in the pericardium, *N* = 2 embryos, 14 images were analyzed, which were obtained from at least six sections per heart).

### 3.4. Epicardium Formation Is Compromised in Pkp2^mt/mt^ Embryos

**Figure 5:**
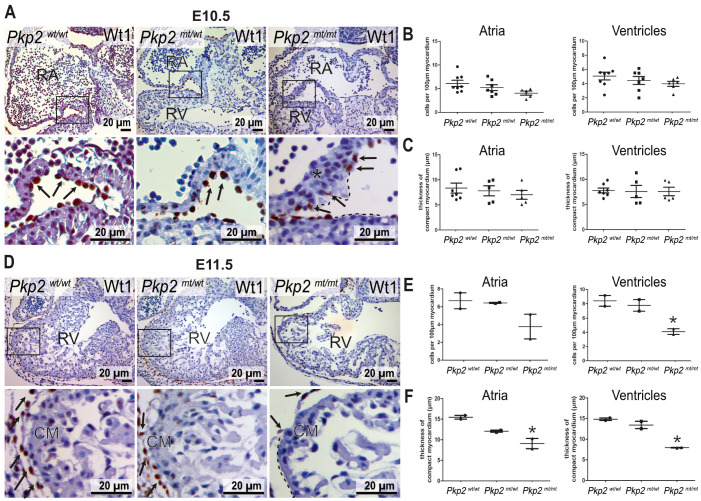
Epicardial coverage and thickness of the myocardium are perturbed in *Pkp2^mt^*^/^*^mt^* hearts. (**A**–**C**) show analyses at E10.5, (**D**–**F**) at E11.5. The left panels (**A**,**D**) present Wt1 immunostaining counterstained with hematoxylin tissue sections of wild-type, *Pkp2^mt^*^/^*^wt^*, and *Pkp2^mt^*^/^*^mt^* hearts at low and high magnification. The black dashed lines mark areas without epicardial coverage, and the asterisk demarcates regions with disorganized cardiomyocytes in the *Pkp2^mt^*^/^*^mt^* samples. Arrows point to epicardial cells. (**B**,**E**) show the results of quantifying the number of Wt1^+^ epicardial cells per 100 µm of myocardial surface. (**C**,**F**) present measurements of compact myocardial thickness. Note the significantly reduced epicardial coverage of ventricles and the reduced myocardial thickness of atria and ventricles at E11.5 in *Pkp2^mt^*^/^*^mt^* hearts. Each data point on the graphs represents the average of measurements or counts normalized to the surface area obtained from one heart. *N* = 5–7 hearts per group in (**B**,**C**); at least three heart sections were analyzed to obtain one data point per animal. *N* = 2 embryos per group in (**E**,**F**); * *p* < 0.05 by one-way ANOVA and Tukey’s multiple comparison test.

Cardiomyocytes may not be the only cell type expressing desmosomal junctions in the heart. Thus, keratin intermediate filament polypeptides, which are known to be anchored to desmosomes in epithelial cells, were detected in the epicardium of quail embryos, and keratin 19 was recently classified as a marker of murine epicardial cells [22,23]. We extended these observations by first performing immunofluorescence microscopy of embryonic hearts. A strong keratin 8 immunosignal was seen exclusively in epicardial cells (Figure S4A). For better resolution, we made use of the previously established homozygous Krt8-YFP mouse producing EYFP-labeled keratin 8 polypeptides from the endogenous keratin 8 gene locus. Here, we were able to detect distinct keratin bundles in the epicardium of the adult heart (Figure S4A). Finally, we studied keratin and desmosomal protein expression in hiPSC-derived epicardial cells. Keratin 8-positive filament bundles were observed together with desmoplakin, desmoglein 2, and Pkp2-positive puncta at cell borders (Figure S4B). Taken together, we concluded that epicardial cells exhibit hallmarks of epithelial differentiation, i.e., an extensive keratin network and desmosomes. We therefore asked whether Pkp2 deficiency impacts the development of epicardial cells. We performed immunostaining for Wilms tumor 1 (Wt1) antigen, which is a marker of epicardial cells during cardiogenesis. In wild-type murine embryonic hearts, epicardial cells cover the entire outer surface of the myocardium, providing a continuous squamous epithelial sheet. However, in *Pkp2^mt^*^/^*^mt^* embryonic hearts, epicardial cells were only loosely attached to the myocardium, and some myocardial areas were devoid of epicardial cell coverage at E10.5 (Figure 5A). To quantify the loss of epicardial coverage, we assessed the number of Wt1^+^ epicardial cells in different regions. At E10.5, epicardial cell coverage was slightly but not significantly reduced in atrial chambers compared to ventricular chambers (Figure 5B). The unexpectedly minor changes may be a consequence of the high proliferation capacity of epicardial cells, which compensates for the regionally reduced number of epicardial cells. Furthermore, the expansion of trabecular myocardium was regionally suppressed in *Pkp2^mt^*^/^*^mt^* samples, whereas the thickness of compact myocardium remained unchanged across different samples at E10.5 (Figure 5C). Assessment of epicardial cell coverage and myocardial thickness on surviving embryos at E11.5 demonstrated a marked reduction in both epicardial cells and myocardial thickness (Figure 5D–F). To find out whether enhanced apoptosis may be involved, we performed anti-cleaved caspase 3 immunohistology. We did not detect positive cells in the epicardium of the mutant hearts, neither in regions with normal epicardial coverage nor in regions with altered epicardium or in aberrant cell clusters that had detached from the heart and were released into the pericardial space (Figure S5). It is therefore safe to conclude that an increase in cell death does not account for the drastic loss of epicardial coverage.

### 3.5. Epicardial Cell Attachment to Cardiomyocytes Is Disrupted in Pkp2^mt/mt^ Embryos

**Figure 6:**
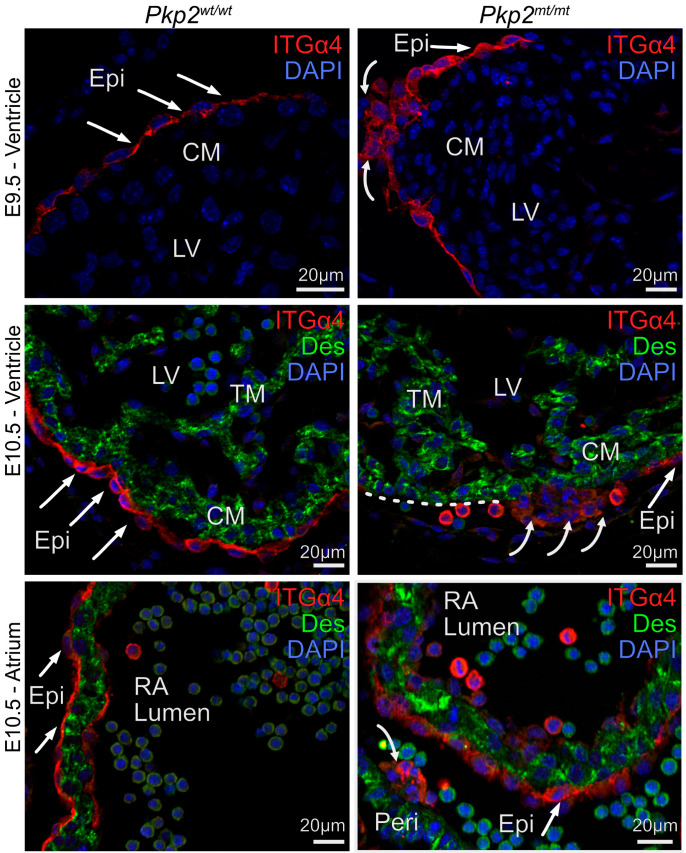
Epicardial cell morphology and attachment to the myocardium are severely perturbed in E9.5 and E10.5 *Pkp2^mt^*^/^*^mt^* embryos. The fluorescence micrographs show the detection of integrin α4 (ITGα4; red) in epicardial cells and desmin (Des; green) in cardiomyocytes after immunofluorescence labeling. Nuclei are stained with DAPI. Note the squamous epithelial morphology of the wild-type epicardium (arrows), whereas epicardial cells are more rounded and form clusters in the *Pkp2^mt^*^/^*^mt^* hearts (curved arrows). Detached ITGα4^+^ cells are present in the pericardial cavity of mutant left ventricle (LV) and right atrium (RA) at E10.5 (curved arrows). Note disrupted cardiomyocyte arrangements in E10.5 ventricle adjacent to a cluster of cells with reduced ITGα4 expression. The dashed white line delineates a region of the myocardial surface that is not covered by epicardial cells. *N* = 3–5 hearts per group, at least 6 heart sections, and 10 images were analyzed per sample. CM, compact myocardium; TM, trabecular myocardium; Epi, epicardium; RA, right atrium; LV, left ventricle.

Proepicardial cells migrate from the liver primordium to the developing heart, where they attach to cardiomyocytes via the adhesion molecule integrin α4 (ITGα4). We therefore examined whether Pkp2 deficiency impacts epicardial attachment and expansion on cardiomyocytes. Starting at E9.5, epicardial cells of *Pkp2^mt^*^/^*^mt^* embryos lost their characteristic thin, squamous epithelial morphology in restricted areas, adopting a rounded, mesenchymal-like shape. They often formed multiple cell layers. ITGα4 expression was reduced (Figure 6). Reduced epicardial attachment to the myocardium persisted at E10.5. At this stage, myocardial regions without epicardial coverage had visibly expanded in the atrium and ventricle (Figure 6). Notably, ITGα4^+^ cells were still observed in the pericardial cavity, suggesting that epicardial cells were extruded from the tissue.

### 3.6. Epicardial Cells Transit into Hematopoietic Stem Cells

**Figure 7:**
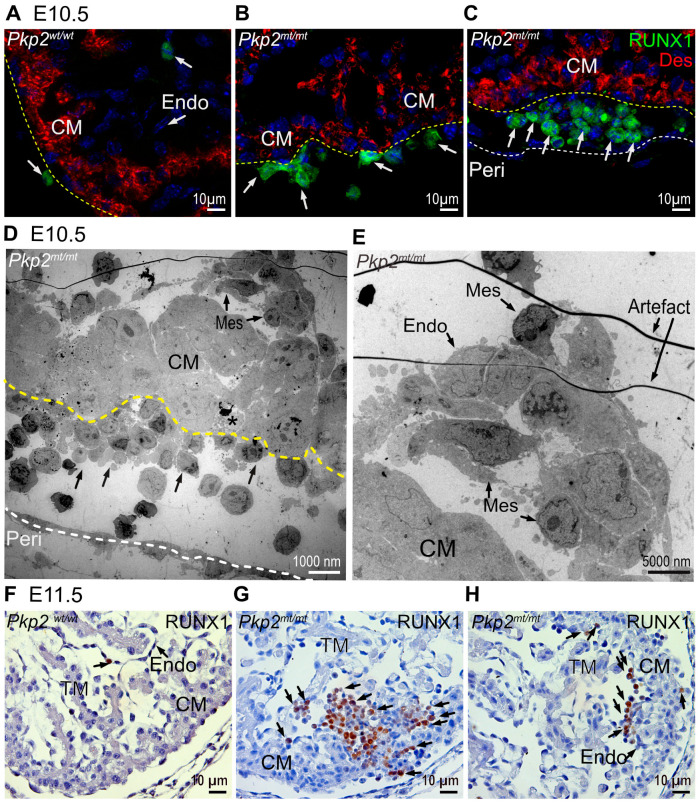
RUNX1^+^ hematopoietic cells located in epicardial and endocardial cell layer are increased in *Pkp2^mt^*^/^*^mt^* hearts at E10.5 and E11.5. The images show detection of RUNX1 by immunofluorescence (**A**–**C**) and immunohistology (**F**–**H**) in *Pkp2^wt^*^/^*^wt^* and *Pkp2^mt^*^/^*^mt^* hearts. *N* = 3–5 hearts per group at E10.5; *N* = 2 embryos per group at E11.5. At least 6 heart sections were analyzed per individual heart. Desmin marks the cardiomyocytes, and nuclei are stained with DAPI. The immunohistological sections are counterstained with hematoxylin. Arrows point to RUNX1^+^ cells. Note that the scarce RUNX1^+^ cells in wild-type hearts are attached to the epicardial and endocardial surface (**A**). In contrast, an increased number of RUNX1^+^ cells is detected in *Pkp2^mt^*^/^*^mt^* epicardium and in regions of lost epicardium at E10.5 (**B**,**C**), and among endocardial cells and within the myocardium at E11.5 (**G**,**H**). (**D**) The transmission electron micrograph of *Pkp2^mt^*^/^*^mt^* myocardium confirms the loss of epicardial cells and their replacement by rounded cells that differentiate into erythrocyte-like cells (arrows). Note the disarrangement of adjacent cardiomyocytes. The dashed yellow line marks the border between cardiomyocytes and epicardial derivatives and a dashed white line marks the pericardium A dying cell is marked by an asterisk. (**E**) The electron micrograph shows a magnified region of the top right corner in (**D**). Mesenchymal-like (Mes) endocardial cells are rounded and form a local aggregate in the sub-endocardium. CM, compact myocardium; Endo, endocardium; TM, trabecular myocardium; Peri, pericardial wall.

The loss of the squamous epithelial morphology of epicardial cells suggested that they lose their cellular identity. During development, the heart is a site of de novo hematopoiesis with cells differentiating into RUNX1^+^ hematopoietic stem cells [24,25] (Figure 7A,F). These events are highly restricted: In E10.5 wild-type hearts, RUNX1 could be detected in 2.9% of all epicardial cells. In *Pkp2^mt^*^/^*^mt^* samples, the number of RUNX1^+^ cells was approximately six times higher in the epicardium (17.03%) (Figure 7B,C). Interestingly, the transformation of endocardial cells to RUNX1^+^ was not affected at E10.5 (about 4% in all groups). Electron microscopy micrographs confirmed the presence of erythrocytes in the replacement of epicardial cells and provided evidence for drastic shape changes in epicardial and endocardial cells in *Pkp2^mt^*^/^*^mt^* samples (Figure 7D,E). By E11.5, RUNX1^+^ clusters had also expanded within the endocardial layer and myocardium of *Pkp2^mt^*^/^*^mt^* embryos (Figure 7G,H).

**Figure 8:**
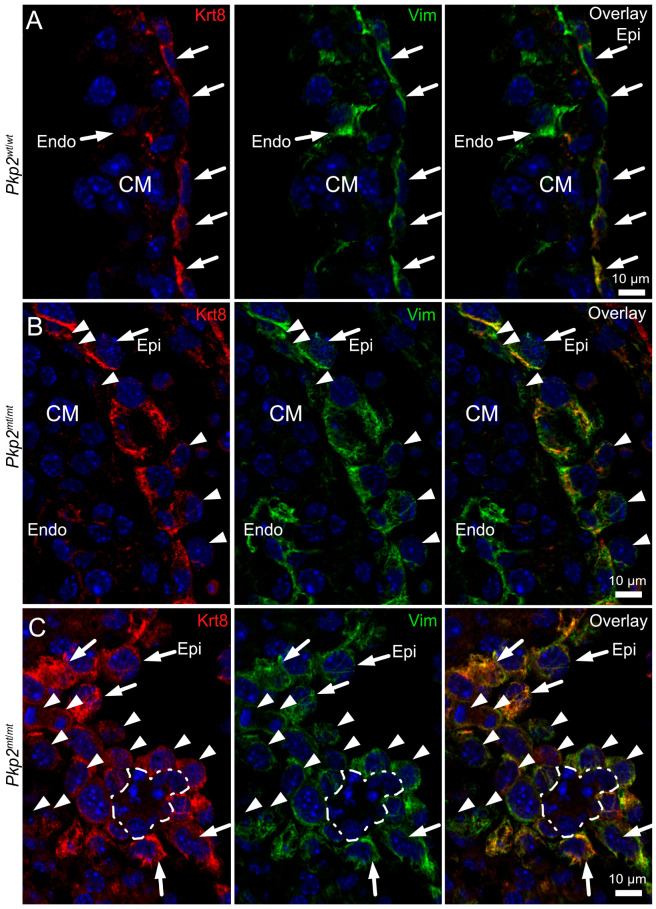
The transition of epicardial cells to mesenchymal and erythropoietic cells is accompanied by changes in intermediate filament protein expression in *Pkp2^mt^*^/^*^mt^* hearts. The immunofluorescence images were recorded at E10.5 in *Pkp2^wt^*^/^*^wt^* (A) and *Pkp2^mt^*^/^*^mt^* hearts (**B**,**C**), detecting keratin 8 (Krt8) and vimentin (Vim). Nuclei were stained with DAPI. (**A**) Note that keratin 8 and vimentin are co-expressed in the squamous epicardial cells (Epi) of the wild-type heart (arrows), whereas endocardial cells (Endo) are only positive for vimentin (arrow). (**B**) shows an immunofluorescence recording of a region in a *Pkp2^mt^*^/^*^mt^* heart where epicardial cells are arranged in two layers. Note that some epicardial cells had migrated into the sub-epicardial space while others face the pericardial space (arrowheads). (**C**) The immunofluorescence micrographs depict a tangential section through the mesenchymally transformed epicardium of a *Pkp2^mt^*^/^*^mt^* heart at the interventricular groove, revealing rounded mesenchymal cells that are strongly positive for vimentin and less positive for keratin (arrowheads). Note also the presence of a cell cluster that is surrounded by these cells and lacks both keratin and vimentin (dashed line). The morphology of these cells differs profoundly from the adjacent mesenchymal, epicardial (arrows), and myocardial cells but is compatible with that of the emergent red blood cell clusters. *N* = 4–6 hearts per group; at least 6 heart sections and 12–18 images were analyzed per sample.

To further validate abnormal mesenchymal transformation of epicardial cells, we investigated the expression of the differentiation markers keratin 8 and vimentin. At E10.5, epicardial cells co-express the epithelial marker keratin 8 and mesenchymal marker vimentin (Figure 8A). The double labeling was also evident in the rounded cells (Figure 8B). Occasionally, in *Pkp2^mt^*^/^*^mt^* epicardium, single cells stained only for vimentin (Figure 8B; arrowheads). This was particularly evident in the interventricular groove, where groups of transformed cells could be detected (Figure 8C). Most conspicuous were clusters of closely spaced cells, which were negative for both vimentin and keratin 8 and surrounded by mesenchymally transformed epicardial cells (dashed line in Figure 8C). The morphology of these cell clusters suggested that they correspond to the red blood cells depicted in Figure 2, Figure 3 and Figure 4. This is in line with the known loss of vimentin during red blood cell differentiation [26,27].

### 3.7. The Ultrastructure of Pkp2^mt/mt^ Hearts Is Severely Perturbed and Provides Evidence for Epithelial-to-Hematopoietic Transition of the Epicardium

**Figure 9:**
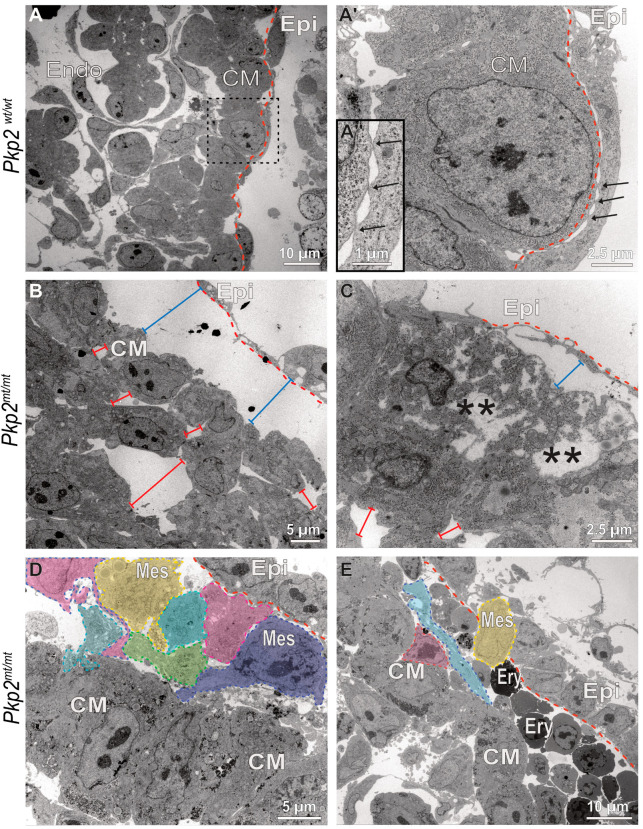
Epicardial cells lose contact with the myocardium, transform into rounded mesenchymal-like cells, and differentiate into erythrocytes. The electron micrographs depict different stages of epicardial-to-hematopoietic transition in *Pkp2^mt^*^/^*^mt^* hearts (**B**–**E**) in comparison to wild-type control hearts (**A**–**A″**) at E10.5. (**A**–**A″**) The pictures (low magnification at left, high magnification of boxed area at right) illustrate the typical arrangement of epicardium (Epi) and myocardium in the *Pkp2^wt^*^/^*^wt^* heart, whereby the elongated flat epicardial cells are coupled to each other via junctional complexes and attach directly to cardiomyocytes at multiple foci, where the plasma membranes of both cell types juxtapose but lack cytoplasmic plaques (arrows; magnified view in (**A″**)). The dashed red line delineates the border between epicardium and myocardium. Note also the columnar arrangement of the junction-linked cardiomyocytes in the compact myocardium (CM). (**B**–**E**) The electron micrographs show that epicardial cells dissociate from the myocardium in *Pkp2^mt^*^/^*^mt^* hearts (bar lines, blue) and that the arrangement of cardiomyocytes is perturbed with increased intercellular spaces (bar lines, red). In some instances, necrotic cardiomyocytes are seen (asterisks in (**C**)). (**D**,**E**) illustrate situations where epicardial cells are arranged in two layers. The outer layer is in continuity with the adjacent, still intact epicardium, whereas the inner layer contains cells with grossly altered shape (colored cells) reflecting mesenchymal transformation (Mes, mesenchymal cell). These cells have reduced cell–cell junctions, they round up and appear to become migratory with processes extending in one direction. These cells seem to undergo further transdifferentiation into erythrocyte-like cells (Ery) that expand in the subepicardial space. *N* = 2 hearts per group. About 200 images were captured from wildtype samples, and about 400 images were captured from the mutant samples. Endo, endocardium.

**Figure 10:**
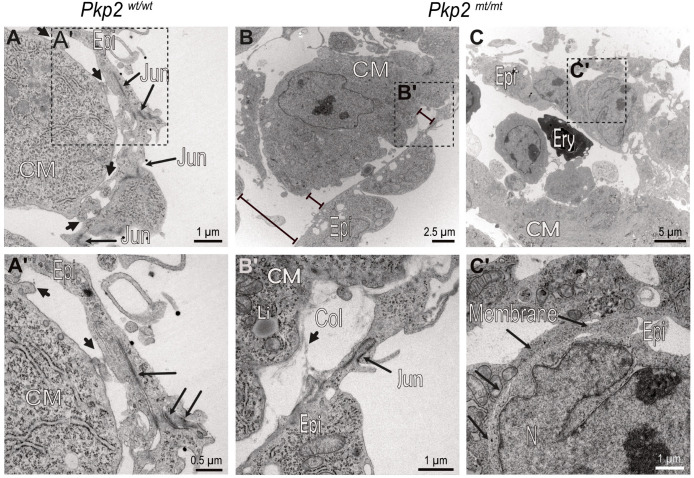
Intercellular epicardial junctions and epicardial–myocardial focal contact points are perturbed in *Pkp2^mt^*^/^*^mt^* hearts. The electron micrographs show details of epicardial and myocardial junctions in E10.5 *Pkp2^wt^*^/^*^wt^* and *Pkp2^mt^*^/^*^mt^* hearts. (**A**,**A′**) Focal contact sites between epicardial cells and cardiomyocytes are marked by short arrows, epicardial intercellular junctions by long arrows. (**A′**) is a magnification of the boxed area in (**A**). (**B**,**B′**) Adhesion of *Pkp2^mt^*^/^*^mt^* epicardial cells to cardiomyocytes is severely compromised, while intercellular junctions are still preserved in this region. Note the distance between epicardium and cardiomyocytes (bar lines). (**B′**) is a magnification of the boxed area in (**B**). (**C**,**C′**) The images depict a region where intercellular epicardial junctions are also compromised. Long arrows in (**C′**) mark junction-free membrane domains of adjacent epicardial cells. (**C′**) is a magnification of the boxed area. *N* = 2 hearts per group. About 200 images were captured from wildtype samples, and about 400 images were captured from the mutant samples. Epi, epicardium; CM, cardiomyocyte; Col, collagen fiber; Jun, intercellular junction; Li, lipid droplet; Ery, erythrocyte; Membrane, plasma membrane.

Wild-type epicardial cells form a one-layered squamous epithelium. Electron microscopy of E10.5 embryos showed that the epicardial cells are connected via prominent junctions and revealed that epicardial cells contact cardiomyocytes at multiple attachment sites, providing focal apposition of the adjacent plasma membranes (Figure 9A,A′ and Figure 10A,A′). The attachment sites are devoid of cytoplasmic plaques (inset in Figure 9A′). The distinct epi-myocardial contacts are lost in E10.5 *Pkp2^mt^*^/^*^mt^* hearts, resulting in dissociation of both cell layers and formation of large gaps (Figure 9B,C and Figure 10B,B′). The gaps occasionally contain remnant collagen fibrils bridging the intercellular space (Figure 10B′). Furthermore, adjacent cardiomyocytes of the compact myocardium lose their cuboidal shape, ordered arrangement, and tight association, becoming more irregular in shape and developing intercellular amorphous spaces (Figure 9B). In some instances, dying cardiomyocytes could be detected (Figure 9C). In contrast, the detached epicardium of *Pkp2^mt^*^/^*^mt^* appeared to be vital in most regions and remained connected through prominent cell–cell junctions similar to the wildtype (compare Figure 10A′ with Figure 10B′). *Pkp2^mt^*^/^*^mt^* epicardial cells that were no longer in contact with the myocardium underwent drastic changes. They first rounded up, gaining a more mesenchymal-like morphology, losing their tight intercellular contact regions (Figure 9D,E and Figure 10C,C′). Instead, they formed elongated cell processes and invaded the adjacent subepicardial space often with the remnant epicardial cell layer on top (Figure 9D,E). Most remarkably, enlarging cell clusters containing typical erythrocytes were observed in the direct neighborhood of these cells (Figure 7D, Figure 9E and Figure 10C). Together, the observations suggest that epicardial cells undergo epithelial-to-hematopoietic transition by first transforming into mesenchymal-like cells, followed by transdifferentiation into hematopoietic cells, which proliferate, giving rise to red blood cells.

## 4. Discussion

The linker protein Pkp2 and transmembrane protein Dsg2 are major desmosomal components that are involved in intermediate filament anchorage and transcellular adhesion, respectively. While loss of Pkp2 abolishes desmosome formation [12], loss of Dsg2 does not completely prevent desmosomal assembly [28,29,30]. Our previous and current findings indicate that loss of Dsg2 and Pkp2 both impair cardiogenesis, albeit through different cellular mechanisms. While deficiency of Dsg2 most likely acts through paracrine mechanisms on desmosome-free endocardial cells at E11.5, resulting in about 30% embryonic lethality at E14.5, depletion of Pkp2 most likely acts directly on desmosome-containing epicardial cells already at E10.5, resulting in 100% embryonic lethality by E12.5. Despite these profound differences, mesenchymal transformation is observed in both scenarios, which gives rise to hematopoietic stem cells. They subsequently develop into red blood cells that accumulate within the myocardium and the pericardial space, which manifests as visible hemopericardium.

**Figure 11:**
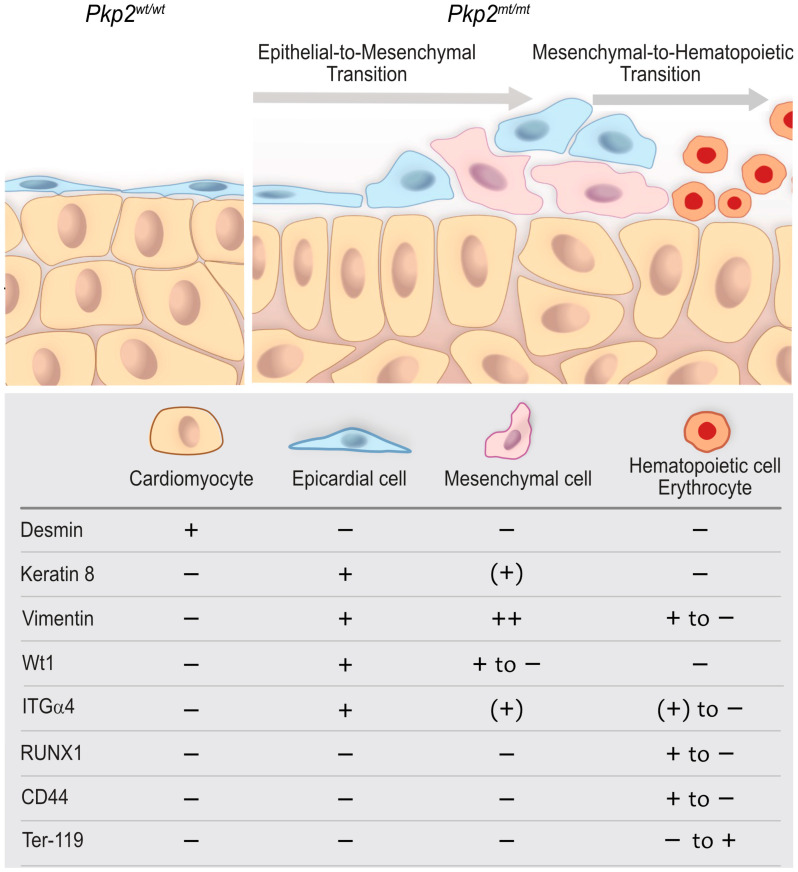
A proposed model that depicts abnormal epicardial development, altered tissue organization, and the emergence of hematopoietic cells in *Pkp2^mt^*^/^*^mt^* hearts compared with wildtype. The scheme highlights the epicardium and compact myocardium interface and the expression of molecular factors in each cell type at E10.5. Wild-type epicardial cells form a squamous, one-layered epithelium that covers the underlying myocardium and is directly attached to cardiomyocytes through multiple focal contact points. In *Pkp2^mt^*^/^*^mt^* hearts, epicardial coverage is compromised. The remaining epicardial cells round up, lose contact with each other, and attain a migratory phenotype indicative of epithelial-to-mesenchymal transition, which is accompanied by downregulation of Wt1, keratin 8, and ITGα4. Subsequent mesenchymal-to-hematopoietic transition is reflected by the formation of hematopoietic stem cells, which are positive for RUNX1 and CD44 but lack keratin 8 altogether. These cells proliferate and give rise to nucleated red blood cells that become Ter-119 positive and completely lose vimentin.

Hemopericardium has not only been reported in transgenic mice with desmosome deficiency [7,9,11] but also in several transgenic mouse models with epicardial deficiency [31,32,33,34]. The underlying cause of hemopericardium remained unclear, though it was attributed to blood leakage into the pericardial cavity due to impaired coronary vessel development or compromised intercellular junctions of cardiomyocytes. Here, we obtained histological evidence that suggests epicardial transformation into mesenchymal cells giving rise to hematopoietic stem cells and blood cells in *Pkp2*-deficient embryonic hearts (Figure 11).

A major remaining question is whether and why epicardial cells successively undergo mesenchymal and hematopoietic differentiation. Despite obtaining extensive histological evidence of lesions at different stages, it is not possible to directly demonstrate that epicardial cells are becoming blood cells based on histological analysis. However, it is well known that epicardial cells contribute to vasculogenesis and angiogenesis [35,36]. Although hematopoietic cells have been identified among proepicardial and epicardial cells [37,38,39], direct transdifferentiation into blood cells has not been documented. Despite their ambiguous origin, hematopoietic cells homing into the epicardium are essential for coronary vasculature development, and they are among the first responders to cardiac injury [37,39,40]. We propose that the absence of desmosomes in the epicardium compromises its ability to maintain full myocardial coverage, coinciding with a differentiation shift toward a mesenchymal fate. Whether this alone directs cells to hematopoiesis or requires additional paracrine factors from the perturbed myocardium remains to be shown. To fully understand the complex sequence of this multistep process, detailed analyses such as cell lineage tracing and single-cell RNA sequencing are needed.

Beyond its barrier function, the epicardium plays a crucial role in heart development both as a reservoir of multipotent progenitor cells and as a promoter of myocardial growth through the secretion of mitogenic signals [17,41,42]. EMT is a key feature of epicardial cells that enables their plasticity and is essential for heart development. Naturally, only a subset of epicardial cells undergoes EMT, while the remaining cells maintain the integrity of the epithelial sheet, which emphasizes the selective and tightly regulated nature of this process. Notably, EMT can also be reactivated in adults experiencing pathological conditions [43,44]. Formation of angioblasts and erythrocytes in the subepicardium of non-vascularised murine embryonic hearts has been documented previously [45], although a direct epicardial transformation was not reported. Our research demonstrates that Pkp2 and desmosomal junctions play an essential role in preserving the epithelial monolayer of the epicardium by restricting its mesenchymal transformation.

In our previous work, we assessed the role of desmosomal proteins in cardiac morphogenesis in mice with a cardiomyocyte-specific deletion of Dsg2 (*Myh6-cre*; *Dsg 2^fl^*^/^*^fl^*) and mice with a constitutive *Dsg2* gene mutation (*Dsg 2*^Δ*E4-E6*^) [11]. Our findings indicated that the lack of desmoglein 2 in *Dsg2*^Δ*E4-E6*/Δ*E4-E6*^ mutants leads to the transformation of endocardial cells into hematopoietic stem cells, which differentiate into erythrocytes. We interpreted this as a result of disrupted crosstalk between cardiomyocytes and endocardial cells [11]. A notable similarity between *Pkp2* mutants and *Dsg2*^Δ*E4-E6*/Δ*E4-E6*^ mutants is that they are characterized by increased hematopoietic stem cells that differentiate into red blood cells. However, significant differences distinguish these two desmosomal protein-deficient models: (i) *Dsg2*^Δ*E4-E4*/Δ*E4-E6*^ mutant hearts still show reduced formation of desmosomes, whereas *Pkp2^mt^*^/^*^mt^* hearts lack them completely. This may explain why Pkp2 depletion has a more detrimental effect on heart development than Dsg2 depletion in terms of phenotypic onset and embryonic lethality. (ii) The primary transforming cell type in *Dsg2*^Δ*E4-E6*/Δ*E4-E6*^ mutants is endocardial, which lacks desmosomes, but probably expresses low levels of Dsg2 mRNA [46]. It remains unclear whether the observed transition in differentiation is driven by paracrine signals from neighboring desmosome-forming cells or reflects a cell-autonomous process mediated by reduced *Dsg2* expression in endocardial cells. If desmosome-dependent, the transition must be triggered by extrinsic mechanical or paracrine signals from the adjacent desmosome-deficient cardiomyocytes. In support of this hypothesis, cardiomyocyte-specific deletion of Dsg2 in the *Myh6-cre*; *Dsg 2^fl^*^/^*^fl^* mice sufficed to transform endocardial cells [11]. In contrast, in *Pkp2^mt^*^/^*^mt^* mutants, mesenchymal transition occurs predominantly in epicardial cells—a cell type that forms desmosomes. This suggests that the increased hematopoiesis in *Pkp2* mutant hearts may be autonomously triggered, independent of or in conjunction with paracrine or mechanical signals from the myocardium. While we cannot distinguish between the different scenarios, we noted disrupted cardiomyocyte organization next to hematopoietic expansions. At this point, we cannot rule out a role for Dsg2 in the phenotype of *Pkp2^mt^*^/^*^mt^* embryonic hearts.

The process of epicardium formation can be divided into two parts: (i) formation of epicardial-myocardial contact points, and (ii) maintenance of the epicardial cell layer. This means while some cells undergo EMT, the rest of the cells maintain their epithelial morphology. Normally, EMT occurs around E12.5 in mice, but in *Pkp2* mutants, epicardial cells initiate EMT as early as E9.5, significantly ahead of the expected timeline. Immunohistochemical and electron micrographs suggest that the abnormality is initiated by a failure in the adhesion of epicardium to cardiomyocytes, possibly due to inefficient adhesion by integrin α4 to VCAM-1. The focal contact sites may correspond to regions where these transmembrane molecules can directly interact. Importantly, it has been suggested that the interaction of VCAM-1 and integrin α4 secure epithelial cell fate by inhibition of TGFβ-induced EMT [47]. In support, we observed that epicardial cells lose their focal contact points with the myocardium in *Pkp2^mt^*^/^*^mt^* hearts (Figure 9 and Figure 10). It is interesting in this context that the knockdown of Pkp2 in primary epicardial cells of neonatal rats showed enhanced cell migration velocity and increased cell proliferation [48].

The epicardial cells undergoing EMT can give rise to multiple cell types. Interestingly, the epicardium-derived cells in *Pkp2* mutants adopt primarily a hematopoietic fate, and do not differentiate into cardiac fibroblasts, as is the case in fetal hearts. This suggests that the fate of epicardium-derived cells depends on different stimuli, potentially involving biochemical and mechanical cues [49,50]. It is possible that both cardiomyocytes and epicardial cells are immature at E10.5 and therefore not capable of differentiating into cardiac fibroblasts. The blood-filled pericardial cavities in *Pkp2* mutant hearts suggest that the transition of epi/endocardial cells to a hemogenic fate may be a common consequence of perturbed desmosome formation during cardiogenesis [11].

## 5. Conclusions

There are two key reasons why understanding the role of desmosome deficiency in early cardiogenesis is critical. First, the embryonic heart serves as a simplified model with fewer interacting cell types, making it an ideal system for dissecting molecular signaling pathways in a less complex context. Second, disruptions in molecular signaling during embryonic development may have lasting effects that re-emerge in the adult heart, though their consequences can vary significantly due to the fundamental differences in cellular plasticity and regenerative capacity between embryonic and adult cardiac tissue. We observed that *Pkp2* heterozygous embryos show abnormal tissue homeostasis. Despite this, they overcome growth and developmental dysfunctions. The abnormality in tissue homeostasis, however, re-emerges during cardiac overload in the adult, leading to arrhythmogenic cardiomyopathy-like symptoms as has been shown for plakoglobin and *Dsg2* mutants [51,52].

## Figures and Tables

**Table 1:** Murine embryos across different gestational ages used for immunohistological and electron microscopy analysis. Resorbed embryos or samples damaged during the harvest and fixation procedure were excluded from the histological analysis.

	E9.5			E10.5			E11.5			E12.5		
Number of litters	3			4			3			3		
Total number of embryos	19			40			22			28		
Genotypes	WT	*PKP2^mt^* ^/*wt*^	*PKP2^mt^* ^/*mt*^	WT	*PKP2^mt^* ^/*wt*^	*PKP2^mt^* ^/*mt*^	WT	*PKP2^mt^* ^/*wt*^	*PKP2^mt^* ^/*mt*^	WT	*PKP2^mt^* ^/*wt*^	*PKP2^mt^* ^/*mt*^
(*n*)	3	10	6	13	16	11	4	8	10	14	12	2
Number of histologically assessed embryos	2	8	3	9	7	10	4	8	2	10	7	0
Dead	0	0	0	0	0	0	0	0	9	0	0	2

## Data Availability

The original contributions presented in this study are included in the article/Supplementary Materials. Further inquiries can be directed to the corresponding author.

## Supplementary Materials

The supporting information can be downloaded at: https://www.mdpi.com/article/10.3390/cells14221751/s1. Table S1. List of primary and secondary antibodies used in immunohisto/cyto chemistry. Figure S1. Generation of Pkp2 mutant mice. Figure S2. *Pkp2^mt^*^/^*^mt^* myocardium lacks bona fide desmosomes. Figure S3. The expression pattern of N-cadherin is not dependent on Pkp2. Figure S4. Keratin intermediate filaments and desmosomes are hallmark features of epicardial cells. Figure S5. Detection of apoptosis by immunostaining for cleaved caspase-3 at E10.5.

## Abbreviations

The following abbreviations are used in this manuscript:

EMT	Epithelial to mesenchymal transition
