# Supplementary material for: Epicardial Abnormalities and Mesenchymal/Hematopoietic Cell Expansion in Plakophilin 2-Null Mouse Embryonic Hearts
Source: Cells. 2025 Nov 8;14(22):1751. doi: 10.3390/cells14221751 (PMC12651295; doi:10.3390/cells14221751)
Supplement: Supplementary file 1 [file cells-14-01751-s001.zip › cells-3662222-supplementary.pdf]

Table S1. List of primary and secondary antibodies used in immunohisto/cyto chemistry

Primary Antibody				
**Antigen**	**Host Species**	**Supplier (Specifications)**	**Working Dilution**	**Secondary Antibody**
Plakophilin 2	Guinea Pig	Progen (GP-PP2)	1:100	Anti-guinea pig (Alexa 488)
Desmoplakin 1 (C-terminus)	Guinea Pig	Progen (DP-1)	1:500	Anti-rabbit IgG (Alexa 555)
Desmoglein 2	Rabbit	Own production*	1:500	Anti-rabbit IgG (Alexa 488 and Dylight 550)
Desmin	Mouse	Sigma, (clone DE-U-10)	1:200	Anti-mouse IgG (Alexa 555)
RUNX1	Rabbit	Abcam, (Ab92336)	1:500	Anti-rabbit IgG (Dylight 555)
N-cadherin	Rabbit	Abcam, (EPR19658)	1:500	Anti-rabbit IgG (Alexa 488 and Dylight 555)
CD49d/Integrina4	Rabbit	Invitrogen (RM268)	1:500	Anti-rabbit IgG (Dylight 550)
Wt-1	Rabbit	Invitrogen (6L9X6)	1:1400	ZytoChem Plus (HRP) Polymer Kit
CD31	Rat	Dianova (clone SZ31)	1:500	Anti-Rat IgG (Alexa 555)
CD44	Rat	BD Pharmingen (clone IM7)	1:500	Anti-Rat IgG (Alexa 555)
Keratin 8	Rat	Developmental Studies Hybridoma Bank (TROMA-I)	1:10	Anti-Rat IgG (Alexa 555)
Vimentin	Guinea Pig	Progen	1:500	Anti-guinea pig (Alexa 488)
Cleaved Caspase-3	Rabbit	BioAcademica	1:1000	ZytoChem Plus (HRP) Polymer Kit

* N. Schlegel et al., Desmoglein 2-mediated adhesion is required for intestinal epithelial barrier integrity. Am J Physiol Gastrointest Liver Physiol 298, G774-783 (2010)

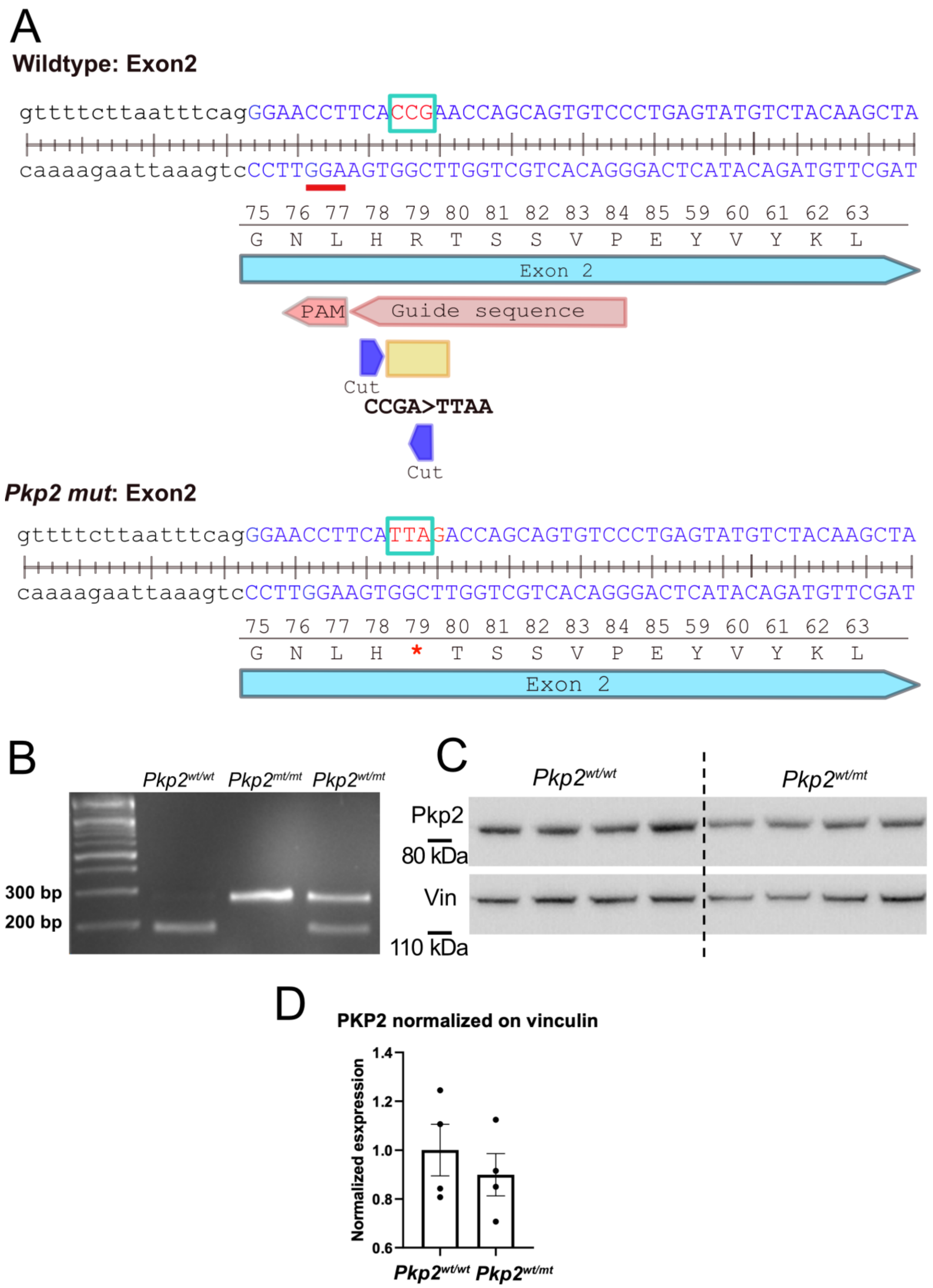

A
Wildtype: Exon2
gttttcttaatttcagGGAACCTTCACCGAACCAGCAGTGTCCCTGAGTATGTCTACAAGCTA
caaaagaattaaagtcCCTTGGAAGTGGCTTGGTCGTCACAGGGACTCATACAGATGTTCGAT
75 76 77 78 79 80 81 82 83 84 85 59 60 61 62 63
G N L H R T S S V P E Y V Y K L
Exon 2
PAM
Guide sequence
Cut
CCGA>TTAA
Cut
Pkp2 mut: Exon2
gttttcttaatttcagGGAACCTTCATTAGACCAGCAGTGTCCCTGAGTATGTCTACAAGCTA
caaaagaattaaagtcCCTTGGAAGTGGCTTGGTCGTCACAGGGACTCATACAGATGTTCGAT
75 76 77 78 79 80 81 82 83 84 85 59 60 61 62 63
G N L H * T S S V P E Y V Y K L
Exon 2
B
Pkp2wt/wt
Pkp2mt/mt
Pkp2wt/mt
300 bp
200 bp
C
Pkp2wt/wt
Pkp2wt/mt
Pkp2
80 kDa
Vin
110 kDa
D
PKP2 normalized on vinculin
Normalized esxpression
1.4
1.2
1.0
0.8
0.6
Pkp2wt/wt
Pkp2wt/mt

Figure S1. Generation of *Pkp2* mutant mice. **A)** The schematic illustrates the design of the mutation that was introduced into exon 2 of the *Pkp2* gene using CRISPR/Cas technology. The resulting stop codon truncates the encoded Pkp2 polypeptide at amino acid 78. **B)** The pictures shows ethidium bromide-stained PCR products after agarose gel electrophoresis obtained from embryonic biopsy samples. Note that only the mutant ~300 bp fragment can be detected in the $Pkp2^{mt/mt}$ sample, whereas the mutant and ~200 bp wild-type fragment are present in the $Pkp2^{wt/mt}$ sample and the wild type fragment is exclusively amplified from the $Pkp2^{wt/wt}$ sample. **C)** The immunoblots detects of Pkp2 and vinculin (Vin) in wild-type and heterozygous adult mouse hearts. D) shows a histogram of the quantification of Pkp2 protein expression normalized to that of vinculin revealing a trend toward Pkp2 reduction in $Pkp2^{wt/mt}$ (n=4).

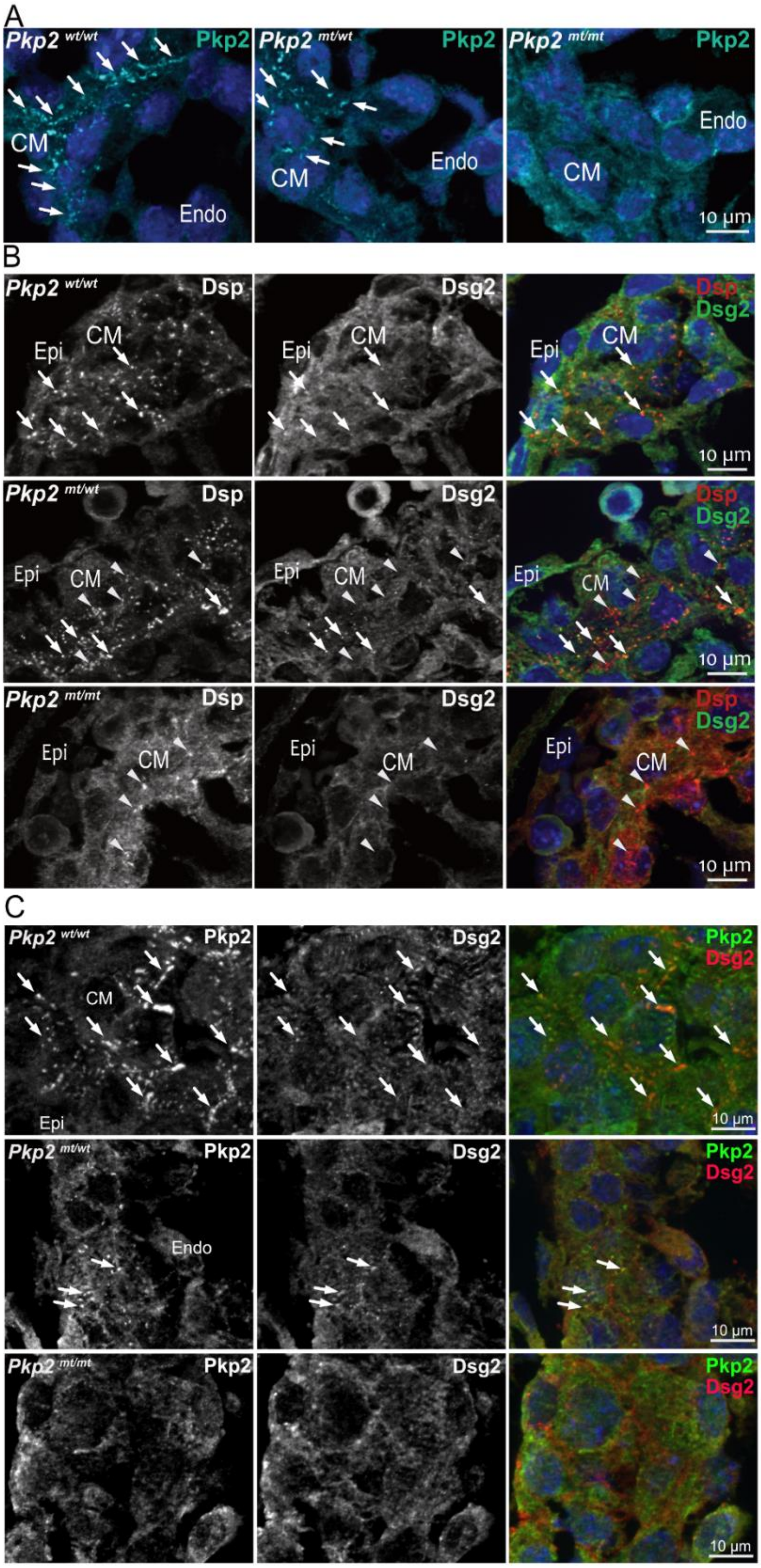
A
Pkp2 wt/wt
Pkp2
CM
Endo
Pkp2 mt/wt
Pkp2
CM
Endo
Pkp2 mt/mt
Pkp2
Endo
CM
10 µm
B
Pkp2 wt/wt
Dsp
Epi
CM
Dsg2
Epi
CM
Dsp
Dsg2
Epi
CM
10 µm
Pkp2 mt/wt
Dsp
Epi
CM
Dsg2
Epi
CM
Dsp
Dsg2
Epi
CM
10 µm
Pkp2 mt/mt
Dsp
Epi
CM
Dsg2
Epi
CM
Dsp
Dsg2
Epi
CM
10 µm
C
Pkp2 wt/wt
Pkp2
CM
Epi
Dsg2
Pkp2
Dsg2
10 µm
Pkp2 mt/wt
Pkp2
Endo
Dsg2
Pkp2
Dsg2
10 µm
Pkp2 mt/mt
Pkp2
Dsg2
Pkp2
Dsg2
10 µm

Figure S2. *Pkp2*$^{mt/mt}$ myocardium lacks bona fide desmosomes. Desmosomal components are detected by immunofluorescence microscopy in E9.5 ventricular myocardium. **A)** The images show that anti-Pkp2 fluorescence localizes to distinct puncta (arrows) at cell borders of adjacent cardiomyocytes in wild-type myocardium. The PKP2 puncta are reduced in the heterozygous *Pkp2*$^{mt/wt}$ sample and completely absent in the homozygous *Pkp2*$^{mt/mt}$ heart. Nuclei are labeled with DAPI (blue). **B)** The double immunofluorescence micrographs reveal that anti-desmoplakin (Dsp)-fluorescence (left panel) and anti-desmoglein 2 (Dsg2) fluorescence (middle panel; overlay at right) colocalize at some defined plasma membrane sites (arrows) in the wild-type and *Pkp2*$^{mt/wt}$ myocardium but not in the *Pkp2*$^{mt/mt}$ myocardium at E9.5. Arrowheads point to puncta that are only positive for Dsp. **C)** The double immunofluorescence micrographs reveal that anti-plakophilin 2 (Pkp2)-fluorescence (left panel) and anti-desmoglein 2 (Dsg2) fluorescence (middle panel; overlay at right) colocalize at some defined plasma membrane sites (arrows) in the wild-type and *Pkp2*$^{mt/wt}$ myocardium but not in the *Pkp2*$^{mt/mt}$ myocardium at E10.5. CM, compact myocardium; Endo, endocardium; Epi, epicardium.

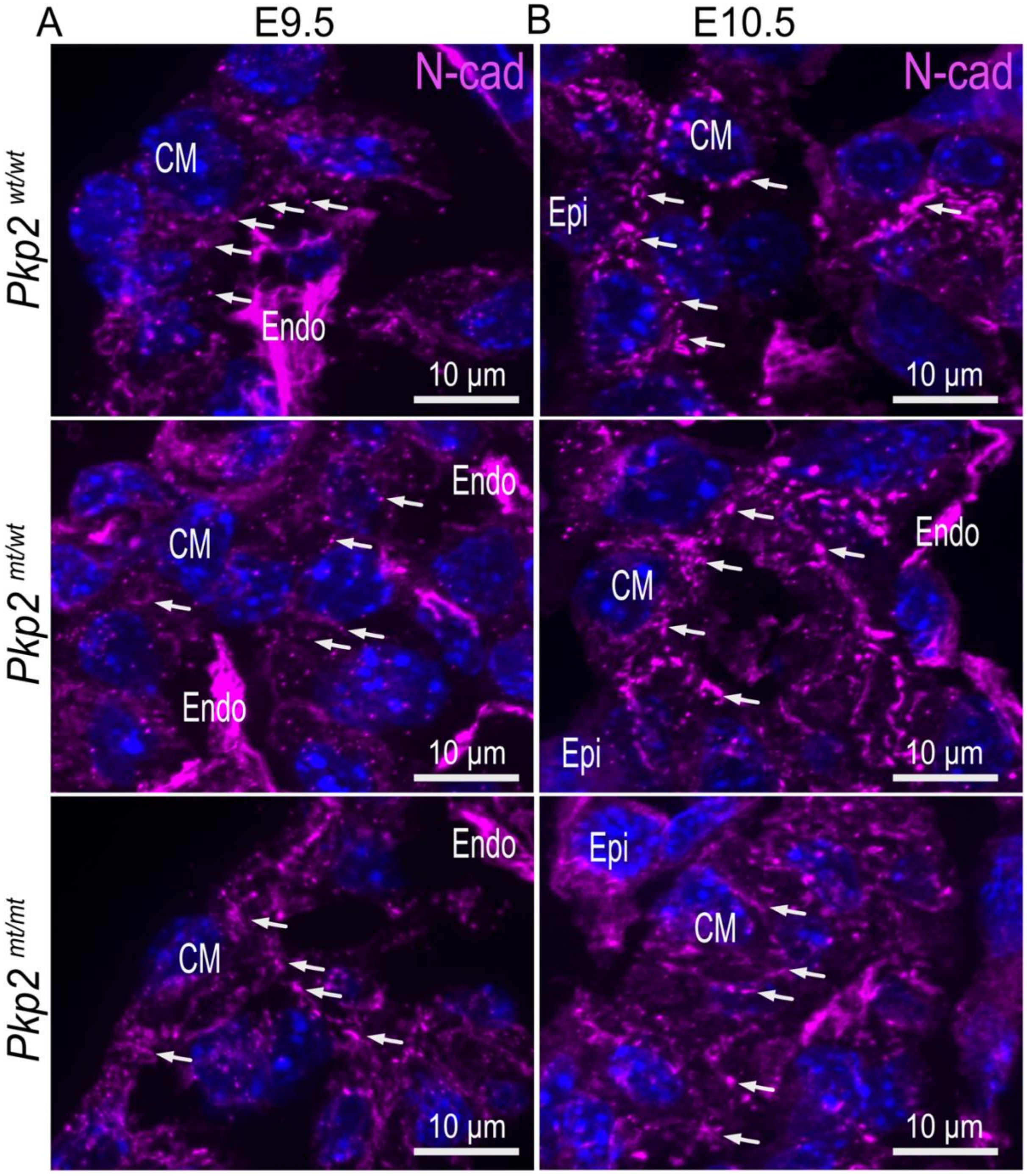

A
E9.5
B
E10.5
N-cad
N-cad
Pkp2 wt/wt
Pkp2 mt/wt
Pkp2 mt/mt
CM
Endo
Epi
10 µm

Figure S3. The expression pattern of N-cadherin is not dependent on Pkp2. The pictures show immunostaining for N-cadherin (N-cad) in embryonic ventricular tissue at E9.5 (**A)** and E10.5 **(B)**. Nuclei are detected by DAPI (blue). Note the punctate staining *Pkp2*$^{wt/wt}$, *Pkp2*$^{wt/mt}$ and *Pkp2*$^{mt/mt}$ hearts (arrows), which becomes more pronounced at E10.5. CM, compact myocardium; Endo, endocardium; Epi, epicardium

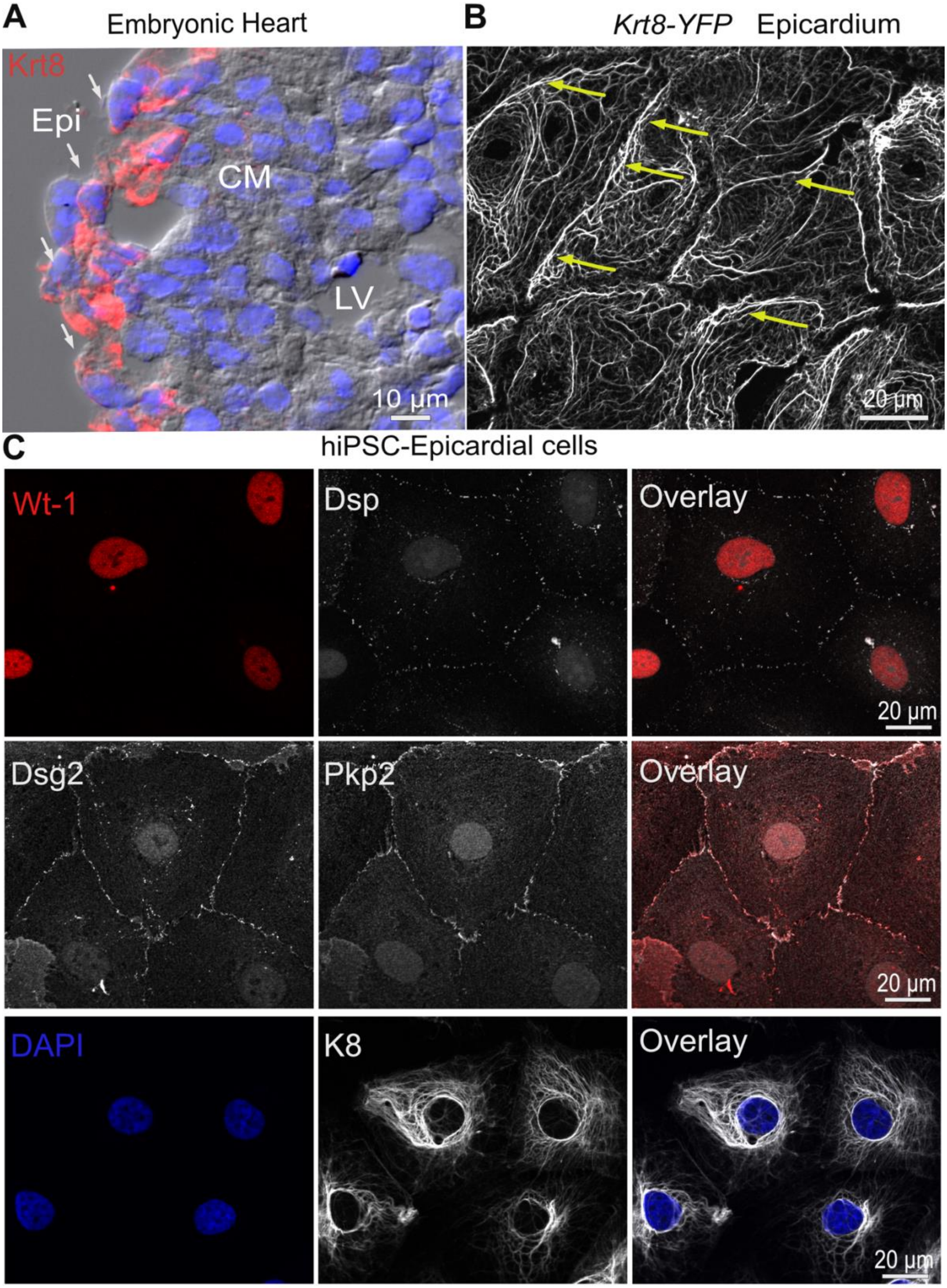
A
Embryonic Heart
Krt8
Epi
CM
LV
10 µm
B
Krt8-YFP Epicardium
20 µm
C
hiPSC-Epicardial cells
Wt-1
Dsp
Overlay
20 µm
Dsg2
Pkp2
Overlay
20 µm
DAPI
K8
Overlay
20 µm

Figure S4. Keratin intermediate filaments and desmosomes are hallmark features of epicardial cells. **A)** The overlay of anti-keratin 8 immunofluorescence (red), DAPI-labeled nuclei (blue) and corresponding phase contrast images reveals selective expression of keratin 8 in epicardial cells (Epi; arrows) of an E11.5 wild-type heart. **B)** Fluorescence microscopy detecting Krt8-YFP in adult epicardial cells that had been isolated from a *Krt8-YFP* transgenic mouse. Note the presence of an extended cytoplasmic network with pronounced keratin bundles (yellow arrows). **C)** The fluorescence images were obtained from hiPSC-derived epicardial cells after staining for the epicardial marker Wt-1 together with the desmosomal component desmoplakin (Dsp) (top panel), after double immunolabeling for the desmosomal proteins desmoglein 2 (Dsg2) and plakophilin 2 (Pkp2) (middle panel) and detection of keratin 8 together with nuclear DAPI staining (lower panel). Single fluorescence micrographs are shown at left and in the middle, merged pictures at right.

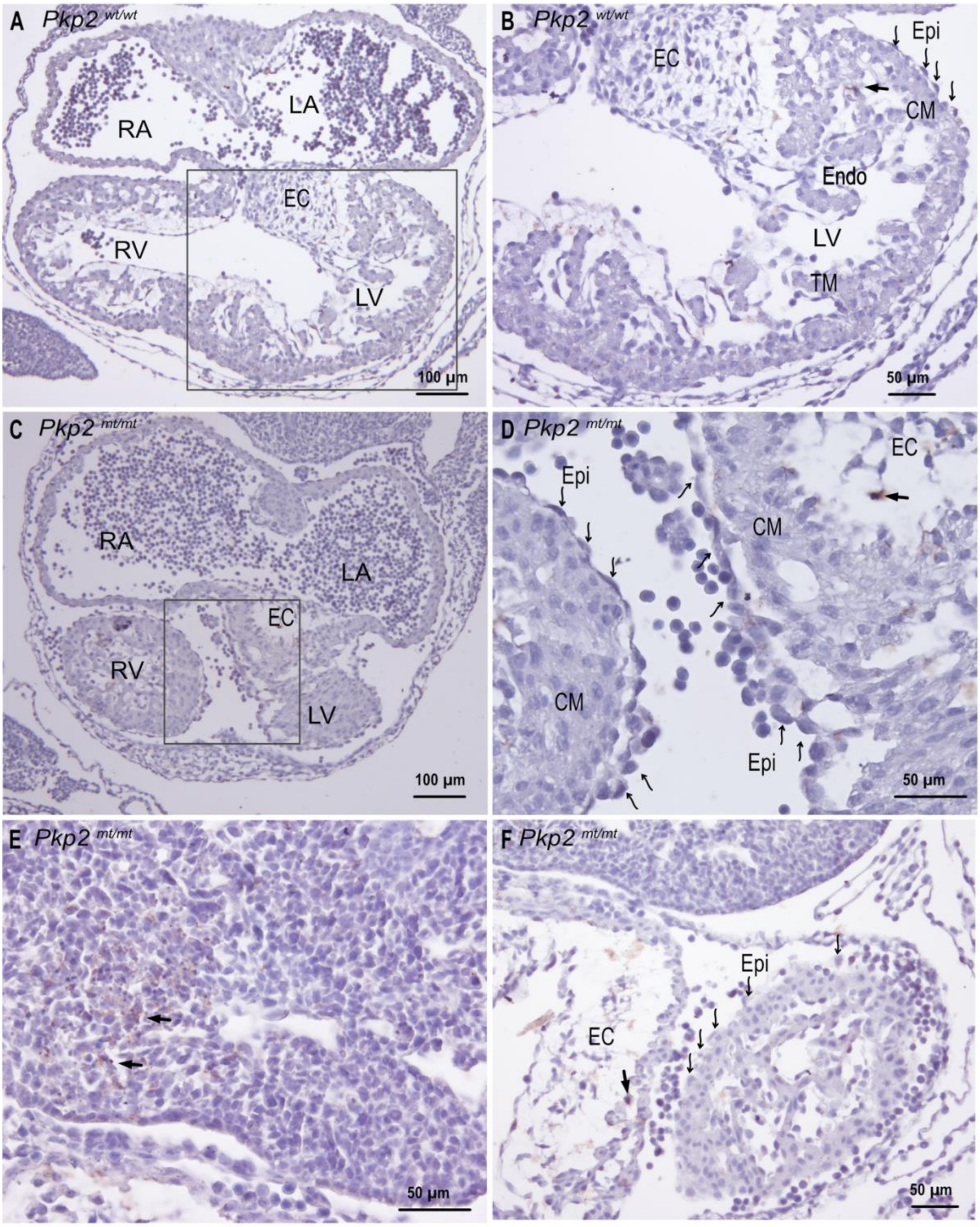

A Pkp2 wt/wt
RA
LA
EC
RV
LV
100 µm
B Pkp2 wt/wt
EC
Epi
CM
Endo
LV
TM
50 µm
C Pkp2 mt/mt
RA
LA
EC
RV
LV
100 µm
D Pkp2 mt/mt
EC
Epi
CM
CM
Epi
50 µm
E Pkp2 mt/mt
50 µm
F Pkp2 mt/mt
Epi
EC
50 µm

Figure S5. Detection of apoptosis by immunostaining for cleaved caspase-3 at E10.5. **A-B)** Apoptotic cells are rare in *Pkp2*$^{wt/wt}$ (arrow). **C-D)** Apoptotic cells are not detected in the epicardial cell layer of *Pkp2*$^{mt/mt}$ hearts (curved arrows) that lost their attachment to cardiomyocytes or changed their epithelial morphology. Adjacent rounded cells and erythrocytes are also not undergoing apoptosis. Furthermore, cell clusters that are present in the pericardial space are non-apoptotic. Arrow points to a cleaved caspase-3 signal in the endocardial cushion (EC) in B, D and F. **E)** Positive control of cleaved caspase-3 staining in *Pkp2*$^{mt/mt}$ embryonic tissue. The boxed areas in A and C are shown in B and D, respectively. CM, compact myocardium; Endo, endocardium; Epi, epicardium; TM, trabecular myocardium; LV, left ventricle; RV, right ventricle; LA, left atrium; RA; right atrium.
